# Elicitors of severe allergic reactions – reports from allergists and emergency doctors 

**DOI:** 10.5414/ALX01347E

**Published:** 2017-08-04

**Authors:** M. Hohenadel, K. Beyer, S. Hompes, M. Worm

**Affiliations:** Klinik für Dermatologie und Allergologie, Charité – Universitätsmedizin Berlin, Germany

**Keywords:** severe allergic reactions, elicitors, allergists, emergency operation

## Abstract

Data from the anaphylaxis registry of German-speaking countries indicate that food is the most frequent elicitor of severe allergic reactions in children, insect venom is the most frequent elicitor in adults. The anaphylaxis registry considers data from patients of allergy centers. The aim of the present study was to collect data regarding elicitors, cofactors and the medical care of patients with severe allergic reactions seen by private practice allergists but also patients seen by emergency doctors. From June 2008 to December 2009 70 cases of severe allergic reactions from private practice allergists and 154 from emergency doctors in Berlin were registered. Our data show that the profile of elicitors differs among the reporting groups. The reported causes from allergists were severe reactions to food, insect venom and subcutaneous immunotherapy, the emergency doctors reported insect venom as the most frequent elicitor. Our data show that a systematic evaluation of severe allergic reactions can provide important data about elicitors and circumstances of anaphylaxis. Through a comparison with data from the anaphylaxis registry the analysis of the data from the emergency doctors will allow to determine how many patients with severe allergic reactions are seen by an allergist for further diagnostic work-up and subsequent therapy.

German version published in Allergologie, Vol. 34, No. 2/2011, pp. 60-67

## Introduction 

Anaphylaxis is the most severe form of mast cell-mediated hypersensitivity reaction and, in the worst case, can have a fatal outcome [8, 9]. The most frequent triggers of anaphylaxis include insect venom, food, drugs and latex [10]. 

A prerequisite for the development of IgE-dependent anaphylaxis is a preceding immunologic sensitization. As a result of sensitization B-cells produce IgE-class antibodies. IgE binds to high-affinity IgE-receptors on mast cells and basophilic granulocytes. Upon re-exposure to the allergen the IgE-molecules cross-linking takes place, and mediators (histamine, prostaglandins, leukotrienes, PAF etc.) and cytokines are released [11]. The mediators chemotactically attract neutrophilic granulocytes and thrombocytes that secernate further inflammatory messengers. The clinical picture of anaphylaxis develops: pruritus, erythema, urticaria and/or angioedema, but also gastrointestinal symptoms like nausea, vomiting or diarrhea [11]. Further pathophysiological effects of the mast cell mediators are an increased vessel permeability, vasodilation and bronchospasm. In the worst case this can lead to anaphylactic shock with drop in blood pressure, tachycardia, dyspnea or even asphyxia, unconsciousness or death. An anaphylactic shock can affect all organ systems [15], but the main reasons for fatal outcomes are the impairment of the cardiovascular system due to hypotension following intravascular blood loss as well as a damage to the airways due to bronchoconstriction, hypersecretion and edemas [9]. 

Data on severe allergic reactions are required that take account of elicitors, cofactors and information on patient care. They will help to improve patient education and care [8, 13]. In 2005 the anaphylaxis registry of German-speaking countries was installed. This registry is a web-based questionnaire by which mainly allergy centers in Germany, Austria and Switzerland report cases of anaphylaxis. Until October 2009 there were 1,768 reports by a total of 75 centers. So far, the data have shown that food is the most frequent elicitor in children and insect venom is the most frequent trigger in adults [3]. 

The anaphylaxis registry includes, however, only patients with records in the affiliated centers. Based on the assumption that not each anaphylaxis patient is treated in an allergy center or specialist practice, we have started to collect data from emergency doctors in Berlin and allergists. 

## Methods 

### Reporting of severe allergic reactions by allergists

For this project we developed a standardized questionnaire for the reporting of severe allergic reactions by allergists in private practices. The questionnaire was called “Fragebogen bei Anaphylaxie für die Arztpraxis” (anaphylaxis questionnaire for private practices) and sent by mail or email distribution lists provided by the Verband deutscher Allergologen (Association of German Allergists). The allergists were asked to report “newly diagnosed” patients. 

The structure of the questionnaire resembles the online entry mask of the anaphylaxis registry [3]. The following data were collected: symptoms of the organ systems skin/mucosa, gastrointestinal tract, airways and cardiovascular system; outcome (fatal or non-fatal); location of the event and if the patient had experienced a severe allergic reaction before. In addition, questions on diagnostic work-up, elicitor(s) of the reaction and possible cofactors are addressed. 

The reporting allergists pseudonymize the patients’ names according to the European privacy policy. The reporting practice, the name of the city as well as the federal state are recorded for further inquiry. Pseudonymization is carried out according to the same encoding that is also used in the anaphylaxis registry. This, together with the date of the reaction, allows to identify double entries. 

The questionnaires were also distributed with the help of sales representatives of various manufacturers of allergen extracts. In addition, it was distributed by the regional group chairs of Ärzteverband Deutscher Allergologen e.V. (Medical Association of German Allergists). Furthermore, the questionnaires were distributed at several events on the topic of allergy and anaphylaxis in the Berlin/Brandenburg region. This physical distribution of questionnaires was only for informational purposes so that the physicians were aware of them. 

After the project had been started quarterly newsletters were sent in order to increase the acceptance and motivation of the physicians. Data were collected between July 1, 2008 and December 31, 2009. 

### Reporting of severe allergic reactions by the emergency doctors in Berlin

In November 2007 we started data collection in cooperation with the Berlin fire department and the AG Notarzt Berlin e.V. (Working Group of Emergency Doctors in Berlin). Emergency medical care in Berlin is coordinated by 18 fire departments in the 12 boroughs. The respective emergency doctors had agreed to fill in a questionnaire for each emergency patient with a severe allergic reaction. 

For this purpose we developed a short, comprehensible and well-arranged questionnaire that could be filled in quickly. Data on the reaction, its elicitor(s), cofactors and therapeutic measures were collected. 

The questionnaire was presented to the heads of the fire departments at an informative meeting on the topic of anaphylaxis. In order to promote active cooperation, the heads of the fire departments were reminded of the project by means of a newsletter on previous reports. The data were collected between July 1, 2008 and December 31, 2009. 

## Results 

### Frequent elicitors of severe allergic reactions

A total of 70 cases was reported by the participating 42 private allergy practices. The reports came from 13 of the 16 German federal states. The highest number of reports came from North Rhine-Westphalia (26 cases) and Saxony-Anhalt (13 cases). From each of the other 11 federal states 5 or less cases were reported. In the same period 154 cases were reported by the participating emergency doctors. Reports came from all of the 18 fire departments in Berlin. 

**Figure 1.**
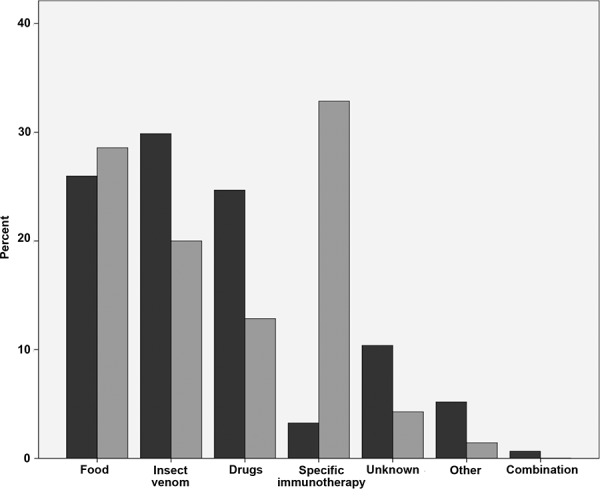
Percentages of elicitors as indicated by emergency doctors (n = 154, dark gray) as compared to allergists (n = 70, light gray).

The most frequent elicitors reported by emergency doctors were insect venom (n = 46, 29.9%), food (n = 40, 26.0%) and drugs (n = 38, 24.7%). The private allergy practices reported specific immunotherapy (SCIT) (n = 23, 32.9%), food (n = 21, 30.0%) and insect venom (n = 14, 20.0%) as the most frequent elicitors (Figure 1). 

**Figure 2.**
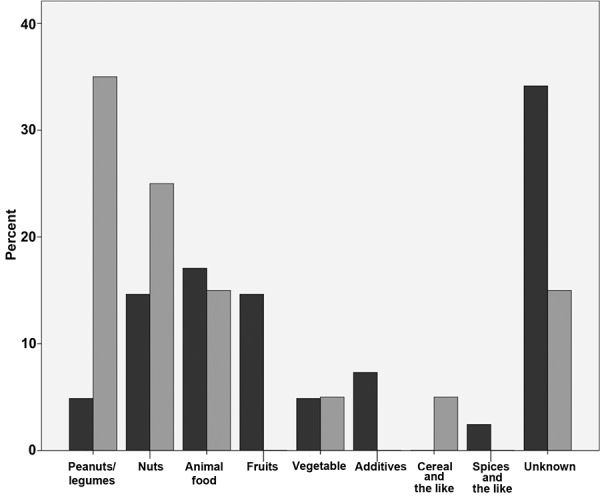
Foods as elicitors of severe allergic reactions as indicated by emergency doctors (n =40, dark gray) and allergists (n = 20, light gray).

From both projects similar food groups have been reported to induce severe allergic reactions: peanuts/legumes, nuts and animal food. Analysis of the data shows that food is frequently suspected to induce severe allergic reactions, but often the exact type of food cannot be determined (Figure 2). 

### Severity of a reaction with regard to its elicitor

The evaluation of the allergic reactions with regard to their severity (according to the classification by Ring and Messmer (Table 1) [12]) shows that in both reporting groups the highest proportion of reported cases was Stage III and only a small percentage of reported reactions lead to cardiac arrest (Table 2). 

**Figure 3.**
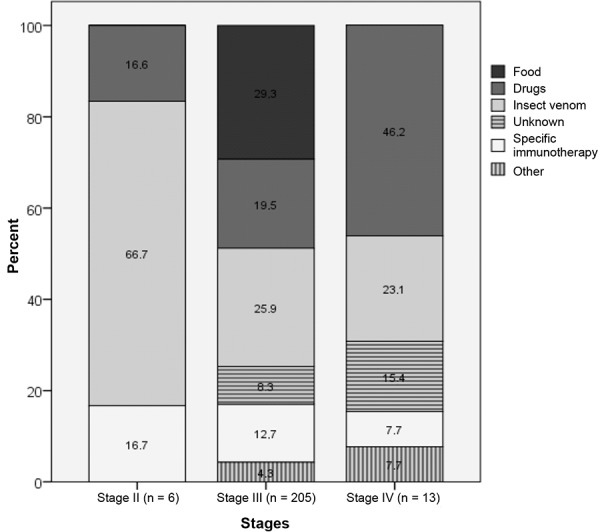
Percentage of elicitors according to the severity of reaction.

Analysis of the elicitors in both reporting groups with regard to the severity of the reaction shows that the most frequently reported elicitor “insect venom” induced Stage II reactions in more than 66% of cases, while “drugs” triggered Stage IV reactions in more than 46% of cases. For the elicitor “food” the most frequently observed type of reaction were Stage III reactions (> 30%) (Figure 3). 

### Severe allergic reactions are age- and gender-related

Analysis according to the age groups “children” (0 – 18 years) and “adults” (> 18 years) shows that relatively more pediatric cases were reported from the private allergy practices. Both allergists and emergency doctors reported more females than males in the group of adults. For children no conclusion on gender-dependence can be drawn due to the low number of cases, but the already known reverse effect compared to the adult group becomes apparent (Table 3). 

## Discussion 

We present first data from an analysis of the reports of severe allergic reactions by allergists and emergency doctors. These first data show that differences exist concerning the frequency of elicitors of severe allergic reactions. This reflects the fact that the analysis includes various groups of patients with severe allergic reactions. The number of 70 reports from the participating practices is still relatively low. Nevertheless, a trend towards subcutaneous immunotherapy (SCIT) being the most frequent elicitor, instead of food and insect venom as in the anaphylaxis registry [3], becomes apparent. 

There can be various reasons for this: first, one could suspect that allergists observe reactions to SCIT rather frequently because they carry out this type of therapy very often; second, it has to be discussed whether in the reported cases specific immunotherapy was carried out according to all safety standards [4]. This question cannot be answered by our investigation. In general, each systemic reaction due to specific immunotherapy should be reported to the Paul-Ehrlich-Institut or at least to the manufacturer. 

Earlier data collected by the Paul-Ehrlich-Institut [5] show that severe allergic reactions during specific subcutaneous immunotherapy are rare. 

In Germany ~ 800,000 new prescriptions of allergen extracts for subcutaneous specific immunotherapy are issued each year. Furthermore, it is likely that the centers participating in the anaphylaxis registry carry out less SCITs per year than private allergy practices. This would be a reason for the lower number of reported severe allergic reactions due to SCIT. In order to analyze this in more detail it would be necessary to collect data on the frequency of SCIT within each of the groups “allergists” and “centers”. 

Furthermore, it has to be taken into account that patients who experience a severe allergic reaction due to, for example, food or insect venom, usually contact an emergency doctor or a first-aid post rather than presenting at an allergy practice. 

Our data underline the importance of collecting data from various health-care levels and demonstrate the influence of demographic parameters, elicitors and cofactors that had already been shown before [1, 16]. 

A weakness of the methods used is the lack of traceability of the distribution of questionnaires. In this context it would be desirable to record exactly to how many and to which practices the questionnaires were sent. In general, our main objective was to increase awareness of this project. For this reason, we have also presented it at various educational events all over Germany. Our medium-term aim is a representative questionnaire response in terms of an anaphylaxis registry including all German federal states by taking into account the number of physicians in each. 

The detailed analysis of the questionnaires shows that the physicians’ reports were partially inexact and that the elicitors of the reaction could not always be determined. In this context it would have to be found out whether the elicitors were really unknown or if an improved diagnostic work-up could increase the number of solved cases. The number of unsolved reasons was > 10% for reporting emergency doctors, but < 5% for private allergy practices. These numbers resemble those from the literature where the frequency of “idiopathic anaphylaxis” is indicated to be 10 – 20% [7, 17]. 

Data reported by the emergency doctors show that for both genders the average age was higher than in other studies. In the study by Decker et al. [2], for example, the average age was 29.3; however, their investigation included reactions that do not involve the respiratory and/or cardiovascular system. This could possibly mean that the probability of an involvement of these two organ systems is also clinically relevant due to age-related changes. This should be investigated in future studies. 

The data presented here confirm results from other studies that also identified food, drugs and insect venom to be the most frequent elicitors of severe allergic reactions [10]. Due to different methodological approaches it is difficult to compare our investigation with other projects. Smit et al. [14] retrospectively analyzed 245 cases of severe allergic reactions in a emergency outpatient clinic in Hong Kong. They demonstrated that food, in this case seafood, was the most frequent elicitor. As in our investigation also in the Hong Kong study, the most frequent elicitors from the group of drugs were non-steroidal antiinflammatory drugs and not, as usually suspected, antibiotics. 

In our emergency doctor project the most frequent elicitor in the pediatric group was SCIT, but the number was still low (n = 8). The low number of children in the reports from emergency doctors could be due to the fact that parents do not call an emergency doctor but are able to render first aid themselves or contact their pediatrician or present at an emergency department. This aspect should be subject to further investigation. A more detailed analysis of those cases in which the elicitor could not be found could offer new insight into the issue of “idiopathic” anaphylaxis. Possibly such investigation could even help to better understand the pathophysiology of anaphylaxis. 

There are only very few studies with allergic practices reporting patients with severe allergic reactions [6, 16] so that it is (almost) impossible to compare our data with existing ones. The reporting group has to be taken into consideration as this can influence the frequency of certain elicitors. 

In conclusion, our data show that elicitors, age and gender distribution differ according to the group of reporting physicians. The data can serve as a basis for the evaluation of the need for action from a medical and health-care point of view. 

## Acknowledgment 

We would like to thank all reporting physicians (allergists and Berlin emergency doctors): Jan Akyeli, Peter Albers, Stefan Altmeyer, Dietrich Andresen, Hans-Richard Arntz, Stephanie Asten, Ulrich Bach, Simone Baumann, Georg Becker, Carola Behling, Jörg Beneker, Michael Benker, Hanka Beutling, Beate Beyer, Wilfried Bohlen, Gerhard Brahm, Jan Breckwoldt, Rita Bunikowski, Hubert Burbach, Marcus Dahlheim, Hermann v.d. Driesch, Konstantinos Drogaris, Mario Eggers, Volker Eissing, Jörg Elster, Christina Erdmann, Gernot Eysselein, Jolanda Faes-Strassl, Wolfram Feußner, Liane Franek, Ines Franke, Karin Funke, Sylviia Gedatus, Thorsten Geyer, Jola Gloza, Raoul Hasert, Heidemarie Heinisch-Krämer, Mario Hensel, Christian Huck, Marcus Joest, Hartmut Kern, Marion Kingl, Sascha Kljucar, Edelgard Knaak, Paul-Otto Kopf, Bernd Krause-Dietering, Franz Krösselhuber, Matthias Krunke, Peter Kuhly, Hendrik Kühne, Petra Kühne, Volker Laag, Matthias Leuschner, Frank Martens, Heike Meier, Jörg Menschikowski, Gerhard Metzner, Norbert Miethke, Ulrike Neise, Katrin Neumann, Petra Neupert, Christine Oeter, Dirk Oldenburg, Beate Petzoldt, Lorenz Reill, Michaele Riehle, Gerolf Ristau, Ottilie Rödder-Wehrmann, Carola Roy, Barum Sarkar, Astrid Schareina, Willi Schmidbauer, Axel Schmidt, Torsten Schröder, Alexander Schuck, Eberhard Sommer, Bernhard Spahn, Sven Tessin, Uta Thieme, Michael Torregroza, Michael Toursarkissian, Jürgen Ungruhe, Anna Vitzthum v. Eckstädt, Andreas Wegner, Wolfgang Wehrmann, Thomas Wiebe, Erika Wulfken, Gudrun Wurl, Wolfgang Zech. 

## Conflict of interest 

The authors have no conflicts of interest. 

**Table 1.** Classification of the severity of anaphylaxis according to Ring and Messmer [12].

**Stage**	**Skin/ ** **mucosa**	**Gastrointestinal tract**	**Airways**	**Cardiovascular ** **system**
I	Pruritus	–	–	–
	Flush			
	Urticaria			
	Angioedema			
II	Pruritus	Nausea	Dyspnea	Tachycardia
	Flush	Cramps	Rhinorrhea^1^	Hypotension
	Urticaria		Hoarseness^1^	
	Angioedema		Arrythmia^1^	
III	Pruritus	Vomiting	Bronchospasm	Shock^2^
	Flush	Defecation^1^	Edema of the larynx^1^	
	Urticaria		Cyanosis^1^	
	Angioedema			
IV	Pruritus	Vomiting	Respiratory arrest	Cardiovascular arrest
	Flush	Defecation^1^		
	Urticaria			
	Angioedema			

^1^not included in our questionnaire; ^2^defined as tachycardia + hypotension + signs of decompensation (collapse/loss of vigilance, dyspnea).

**Table 2.** Number of reported reactions according to their severity and to the gender of the patient.

	**Number of ** **reports**	Stages*			**Gender**	
		**II**	**III**	**IV**	**Female**	**Male**
Emergency doctors	154	5 (3.2%)	140 (90.9%)	9 (5.8%)	87 (56.5%)	61 (39.6%)
Allergists	70	1 (1.4%)	65 (92.9%)	4 (5.7%)	39 (55.7%)	27 (38.6%)
Total	224	6 (2.7%)	205 (91.5%)	13 (5.8%)	126 (56.2%)	88 (39.3%)**

*Stage I reactions have not been included in our evaluation. **For 10 cases (emergency doctors = 6; allergists = 4) the gender of the patient was not indicated; total 4.5%.

**Table 3.** Distribution of children and adults according to gender and age in both projects.

	**Gender**	**Children**	**Age ** **in years ** **(min/max)**	**Mean ** **age ** **(years)**	**Adults**	**Age ** **in years ** **(min/max)**	**Mean ** **age ** **(years)**
Emergency doctors	m/f	5/3	3.0/16.0	10.0/9.2	56/84	20.0/97.0	49.3/53.9
Allergists	m/f	8/6	6.0/17.0	12.9/12.3	19/32	20.0/77.0	41.1/44.1
